# Comparison of Neutralizing Antibody Responses at 6 Months Post Vaccination with BNT162b2 and AZD1222

**DOI:** 10.3390/biomedicines10020338

**Published:** 2022-02-01

**Authors:** Evangelos Terpos, Vangelis Karalis, Ioannis Ntanasis-Stathopoulos, Zoi Evangelakou, Maria Gavriatopoulou, Maria S. Manola, Panagiotis Malandrakis, Despoina D. Gianniou, Efstathios Kastritis, Ioannis P. Trougakos, Meletios A. Dimopoulos

**Affiliations:** 1 Department of Clinical Therapeutics, School of Medicine, National and Kapodistrian University of Athens, 11528 Athens, Greece; johnntanasis@med.uoa.gr (I.N.-S.); mgavria@med.uoa.gr (M.G.); panosmalan@med.uoa.gr (P.M.); ekastritis@med.uoa.gr (E.K.); mdimop@med.uoa.gr (M.A.D.); 2 Section of Pharmaceutical Technology, Department of Pharmacy, School of Health Sciences, National and Kapodistrian University of Athens, 15784 Athens, Greece; vkaralis@pharm.uoa.gr; 3 Department of Cell Biology and Biophysics, Faculty of Biology, National and Kapodistrian University of Athens, 15784 Athens, Greece; zoievag@biol.uoa.gr (Z.E.); mmanola@biol.uoa.gr (M.S.M.); gndespoina@biol.uoa.gr (D.D.G.); itrougakos@biol.uoa.gr (I.P.T.)

**Keywords:** SARS-CoV-2, COVID-19, neutralizing antibodies, humoral immunity, durability, BNT162b2, AZD1222, vaccine

## Abstract

Along with their level of protection against COVID-19, SARS-CoV-2-specific antibodies decline over time following vaccination with BNT162b2. However, relevant data on AZD1222 are scarce. In this context, the aim of this study was to compare SARS-CoV-2 neutralizing antibody (NAb) levels at one, three and six months after second vaccination with the BNT162b2 mRNA vaccine and the ChAdOx1 (AZD1222) viral vector vaccine (NCT04743388). The measurements were performed with the GenScript’s cPassTM SARS-CoV-2 NAbs Detection Kit (GenScript, Inc.; Piscataway, NJ, USA). Overall, data from 282 individuals were included (BNT162b2 *n* = 83, AZD1222 *n* = 199). Both vaccines induced strong NAbs responses at 1 month following vaccination. Interestingly, NAb activity seemed superior with BNT162b2 compared with AZD1222. A gradual decline in NAbs titers was evident at 3 and 6 months post vaccination with both vaccines. However, the superiority of NAb response with BNT162b2 over AZD1222 remained consistent at all time points examined. Furthermore, the elimination rate of the NAb titer was higher throughout during the study period for those vaccinated with AZD1222 compared with BNT162b2. Age, gender, body mass index or comorbidities did not have a significant impact on NAbs levels over time. Our results may inform public health policies regarding the timing of booster COVID-19 vaccine shots.

## 1. Introduction

Vaccines against severe acute respiratory syndrome coronavirus 2 (SARS-CoV-2) have shown significant efficacy in preventing severe disease and coronavirus disease (COVID-19)-associated deaths [1,2,3,4,5,6]. The manufacture process of the approved vaccines is based on two alternative approaches in order to expose the SARS-CoV-2 spike protein to the human immune system [7]. The first one is by using a viral vector such as adenovirus (AZD1222, Ad26.COV2.S), and the second is by applying mRNA technology to induce the antigen expression by human cells (BNT162b2, mRNA-1273) [7]. All vaccines result in robust antibody responses against SARS-CoV-2 [3,4,5,8,9,10]. The approval of several COVID-19 vaccines, which are both safe and effective, is essential in order to ensure vaccine availability worldwide and prevent the short- and long-term impact of COVID-19 [11].

However, it seems that the level of SARS-CoV-2-specific antibodies declines over time, along with the level of protection against COVID-19 [12,13,14,15,16,17]. Importantly, neutralizing antibodies (NAbs) have been associated with immune protection against COVID-19 [18,19]. The emergence of new viral strains has also led to breakthrough infections among vaccinated individuals, especially among those vaccinated several months previously [14,20]. Interestingly, NAbs against the wild-type Wuhan virus strain neutralize new variants of concern including Delta [10]. Nevertheless, this may not be the case for the currently prevalent Omicron variant, although a third booster dose with the BNT162b2 significantly increases the neutralization activity against Omicron [21].

Although the kinetics of antibody response following vaccination with BNT162b2 have been well documented in the literature, relevant data on AZD1222 are scarce. In this context, the aim of this study was to compare SARS-CoV-2 NAbs levels after the second vaccination with the BNT162b2 mRNA vaccine (Pfizer/BioNTech) and the ChAdOx1 (or AZD1222, AZ, Oxford University/AstraZeneca) vaccine.

## 2. Materials and Methods

### 2.1. Clinical Procedure

All subjects participated in a prospective study (NCT04743388) aiming to determine the kinetics of antibody response after vaccination against COVID-19. Following the approval of the study protocol by the Ethics Committee of Alexandra General Hospital (Ref. No. 15/23 December 2020), this study was conducted in accordance with the Declaration of Helsinki and the standards of care of the International Conference on Harmonization for Good Clinical Practice. At the beginning of the study, all subjects provided written informed consent.

The main inclusion criteria were that the participants were eligible for COVID-19 vaccination under the National Immunization Program, were over 18 years of age and could sign an informed consent form. Patients with an active malignant disease, patients taking immunosuppressive medications and patients with end-stage renal disease were not eligible for inclusion. According to the National Immunization Program, the BNT162b2 mRNA vaccine was available to all persons 18 years of age or older at the time of administration, but the ChAdOx1 vaccine was available only to persons 60–64 years of age at baseline. The participants were interviewed for their prior history of COVID-19. Therefore, for comparison, only participants aged 57–67 years who had received the Pfizer vaccine were included in this study in order to have similar ages, i.e., +3 and 3 from the age limits of the vaccine from AZ.

Blood samples were collected on day 1 (D1) before the first vaccination, and then at one (M1), three (M3) and six (M6) months after the second vaccination of each vaccine. The interval between the two administrations of the BNT162b2 mRNA vaccine was 21 days, whereas the corresponding interval for the ChAdOx1 vaccine was 12 weeks (i.e., approximately 3 months), based on the recommendations of the National Immunization Program in Greece. For comparison purposes, all measurements were performed at the same time points following second vaccination, i.e., at M1, M3 and M6.

The confidentiality of the subjects’ data was protected in accordance with the provisions of the General Data Protection Regulation. The identity of all participants was kept absolutely confidential and names were immediately de-identified after sample collection using pseudo-anonymization methods.

### 2.2. Bioanalysis

Within 4 h of blood collection, serum was isolated and stored at −80 °C until the day of measurement. In parallel assays, stored samples from different time points of the same donor were analyzed. Neutralizing SARS-CoV-2 antibodies were measured using an FDA-approved method. The cPassTM SARS-CoV2 NAbs Detection Kit (GenScript, Piscataway, NJ, USA) was used to detect probable SARS-CoV-2 NAbs in blood. The antibody-mediated suppression of SARS-CoV-2 Receptor Binding Domain (RBD) binding to the human host receptor angiotensin converting enzyme type 2 (ACE2) was investigated using this technique.

### 2.3. Data Analysis

In this study, the levels of neutralizing SARS-CoV-2 antibodies were measured 1, 3 and 6 months after the second vaccination with BNT162b2 and ChAdOx1 vaccines. In addition, the average rate of change (RC) between two consecutive time points was calculated based on these values. In the present analysis, RC was calculated for two time intervals, namely M3–M1 (i.e., 60 days) and M6–M3 (i.e., 90 days). The terms RC M3–M1 and RC M6–M3 refer to the ratio (M3–M1)/60 and (M6–M3)/90, respectively. As the inhibition thresholds decrease, RC takes negative values, but for simplicity, the absolute RC values are calculated. It is plausible that the larger the absolute value of the rate of change, the larger the rate of elimination of NAbs. Since the time scale used in this study was “day”, the units of RC are (percentage) inhibitor titers (of SARS-CoV-2 binding per day). In other words, RC refers to the rate of antibody degradation in the body, and negative values are always expected.

The demographic data, concomitant diseases and prescriptions of the participants were collected through personal interviews. Body mass index (BMI) was calculated for each subject based on their weight and height. Subjects were classified into four groups based on their BMI: underweight (BMI less than 18.4), normal weight (BMI 18.5 to 24.9), overweight (BMI 25 to 29.9), and obese (BMI 30 or more).

Statistical analysis began with the calculation of descriptive criteria such as the mean, median, quartiles, and estimation of the dispersion metric. A normality test was performed before statistical comparison between two or more groups. To determine the normality of the data distribution, the Kolmogorov–Smirnov procedure was used. If the nominal normality hypothesis was rejected, it was assumed that the data did not conform to the normal distribution. The data were found to deviate from the normal distribution in all situations in this study, so non-parametric approaches were used for all comparisons. The Mann–Whitney U test was used for comparisons between two independent groups, such as examining sex effect or comparing NAbs levels between the Pfizer and AZ vaccines. The Wilcoxon signed-rank test was used for pairwise group comparisons, e.g., comparing neutralizing antibody levels between two occasions.

The significance level in this study was set to 5% in all cases, and a result was considered significant if the calculated *p*-value (*p*) was less than the significance level. Python v.3.9.2 (Python Software Foundation, Wilmington, DE, USA) was used for statistical analysis.

## 3. Results

### 3.1. Baseline Characteristics

The BNT162b2 and ChAdOx1 vaccines were administered in two doses to 83 and 199 subjects, respectively. Table 1 summarizes the demographic information collected from the study participants. In the Pfizer group (BNT162b2 vaccine), the mean age was 61 years and there were 30 males (36.1%) and 53 females (63.9%). In the AZ group (ChAdOx1 vaccine), 56 men (47.1%) and 63 women (52.9%) participated, while the median age was 61.5 years. The median BMI value was almost the same in both groups (26.7 vs. 26.6), while the BMI distribution was similar in the Pfizer and AZ groups (Table 1). A statistical comparison revealed no significant differences in age and BMI at baseline between the two groups (*p* = 0.957).

### 3.2. Neutralizing Antibody Levels

**Figure 1:**
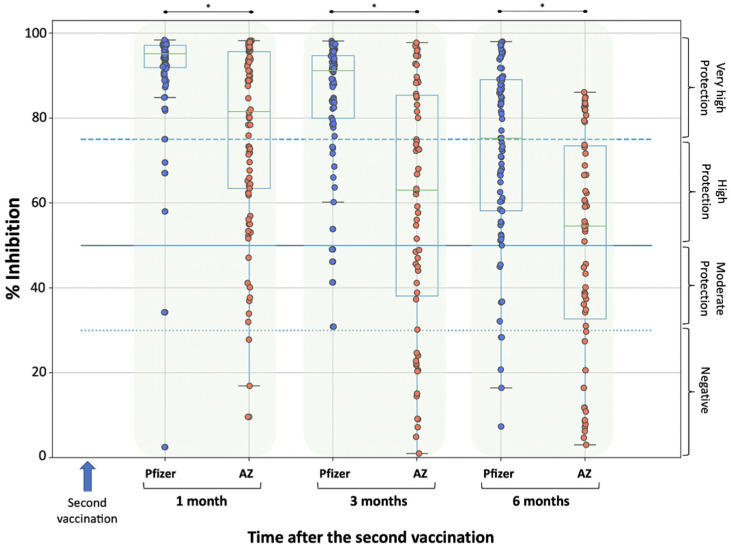
Inhibition (%) of SARS-CoV-2 binding to the human host receptor angiotensin converting enzyme-2 after vaccination with the BNT162b2 (Pfizer) vaccine (83 individuals) and the ChAdOx1 (AstraZeneca, AZ) vaccine (119 individuals). Antibodies were measured on day 1 (vaccination date), one month, three and six months after the second vaccination. Asterisks (*) indicate statistically significant differences between the compared groups (*p*-value < 0.05). The boxplot boundaries show the distribution’s quartiles, whereas the superimposed dots represent individual levels of NAbs inhibition. The dashed lines represent the boundary levels of inhibition, which are 30%, 50% and 75%.

Figure 1 shows the percent inhibition of NAbs at M1, M3 and M6 for volunteers vaccinated with either BNT162b2 or AZD1222. The visual inspection of Figure 1 shows a consistent decline in NAbs levels for both vaccines from one month to six months post vaccination. Pairwise comparisons between M3/M1 and M6/M1 for the BNT162b2 or AZD1222 vaccine revealed statistically significant differences (in all cases the Wilcoxon *p*-value was less than 0.001) explaining the consistent decline in inhibition level.

At baseline, i.e., on the first day of observation (D1), i.e., immediately before vaccination, the percentage inhibition did not differ significantly between the Pfizer and AZ groups (Mann–Whitney *p*-value = 0.298). The median inhibition was 11.97% for the Pfizer group and 15.55% for the AZ group. As there was no difference in NAbs between the Pfizer and AZ groups, comparison at later time points (i.e., M1, M3 and M6) was feasible.

One month after the second vaccination (i.e., at M1), the median inhibitions were found to be significantly different (*p*-value < 0.001), reaching a value of 95.2% and 81.6% in the Pfizer and AZ groups, respectively. At this time, only two subjects (2.4%) receiving Pfizer had NAbs titers less than 50% and were classified as either negative or moderately protected. In the AZ group, 10 volunteers (8.4%) had NAbs levels of less than 50%.

The significantly higher inhibition thresholds for the Pfizer vaccine compared to AZ were also evident at the subsequent time points of M3 and M6; in both cases, *p*-values were less than 0.001. Specifically, three months after the second vaccination, the median inhibition score was 91.25% for those receiving the Pfizer vaccine, compared to the median score of 63.09% for those receiving the AZ vaccine. At this time, the number of individuals in the Pfizer group who were at most moderately protected (NAbs 50% or less) was five (6.0%). The corresponding proportion for participants in the AZ group was 42.0% (i.e., 50 individuals).

Six months after the second vaccination, the median score in the Pfizer group decreased to 75.3%, whereas there was only a small decrease in the AZ group (from 63.09% to 59.4%). Regarding the participants who had received BNT162b2, there were 11 individuals (13.25%) with less than 50% inhibition. Among the participants who had received AZD1222, the corresponding number was 21 (i.e., 17.6%).

**Figure 2:**
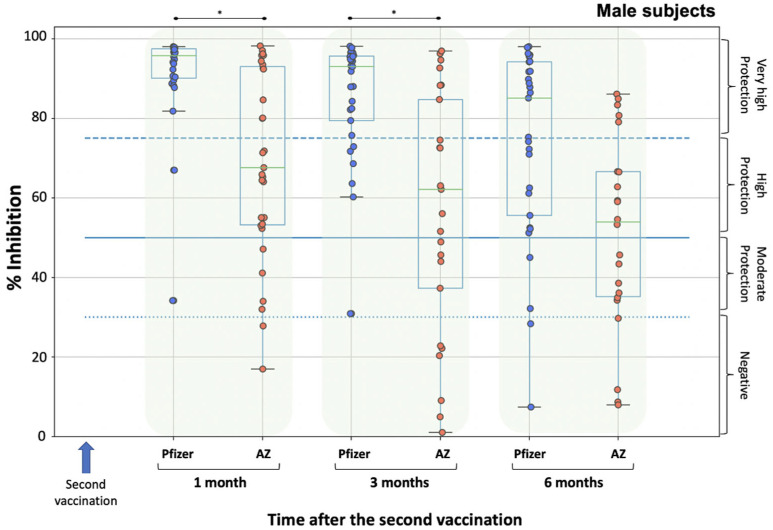
Inhibition (%) of SARS-CoV-2 binding to the human host receptor angiotensin converting enzyme-2 in male subjects after vaccination with BNT162b2 vaccine (Pfizer) and ChAdOx1 vaccine (AstraZeneca, AZ). A number of 30 and 56 men participated in the Pfizer and AZ groups, respectively. Antibodies were measured on day 1 (vaccination date), one month, three months and six months after the second vaccination. Asterisks (*) indicate statistically significant differences between the compared groups (*p*-value < 0.05). The boundaries of the boxplot indicate the quartiles of the distribution, while the overlaid points represent the individual NAbs inhibition values. The dashed lines represent the cut-off values of inhibition, which are 30%, 50% and 75%.

A similar analysis was also performed with respect to gender to determine possible gender differences in the development of the percentage inhibition of SARS-CoV-2 between the two vaccines. First of all, the male subjects in the Pfizer group were compared to the male subjects in the AZ group (Figure 2). No differences were found in males between the two groups at baseline (i.e., D1), allowing comparison at subsequent time points. A statistically significant difference was observed between the Pfizer group and the AZ group at M1 and M3. Male subjects in the Pfizer group had significantly higher scores than those in the AZ group (Figure 2). However, six months (at M6) after the second vaccination, there was no statistical difference in males (*p*-value = 0.096), suggesting that both vaccines have almost similar inhibitory values.

**Figure 3:**
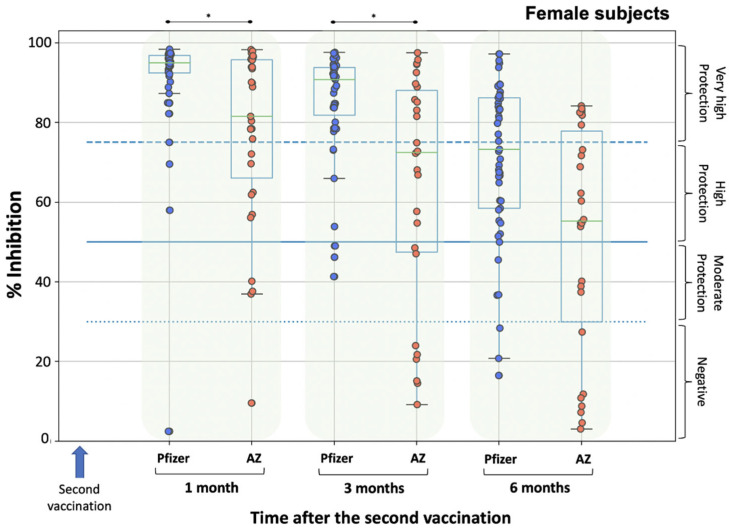
Inhibition (%) of SARS-CoV-2 binding to the human host receptor angiotensin converting enzyme-2 in female subjects after vaccination with BNT162b2 vaccine (Pfizer) and ChAdOx1 vaccine (AstraZeneca, AZ). Fifty-three and sixty-three women participated in the Pfizer and AZ groups, respectively. Antibodies were measured on day 1 (vaccination date), one month, three months and six months after the second vaccination. Asterisks (*) indicate statistically significant differences between the compared groups (*p*-value < 0.05). The boundaries of the boxplot indicate the quartiles of the distribution, while the overlaid points represent the individual NAbs’ inhibition values. The dashed lines represent the cut-off values of inhibition, which are 30%, 50% and 75%.

Furthermore, the female subjects of the Pfizer group were compared with the female subjects of the AZ group (Figure 3). No differences were found in females between the two groups at baseline (i.e., D1), allowing comparison at subsequent time points. A similar pattern to males was observed in females (Figure 3). At M1 and M3, women from the Pfizer group had significantly higher levels than those from the AZ group (*p*-values < 0.001). At M6, women from the Pfizer and AZ groups had similar inhibition titers against SARS-CoV-2 (*p*-value = 0.944).

**Figure 4:**
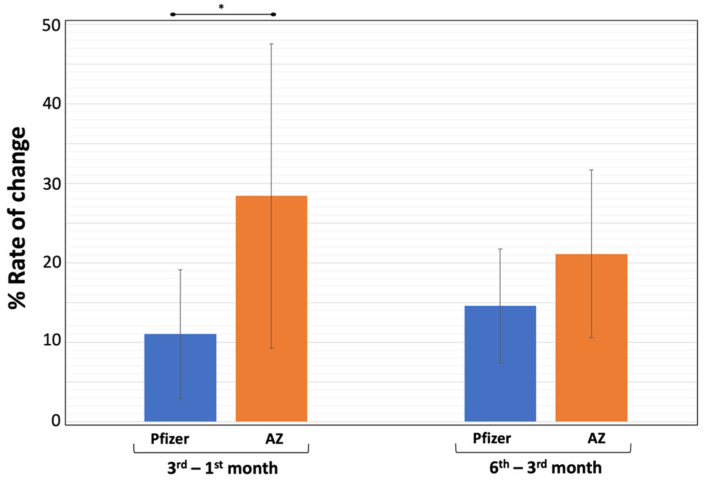
Rate of change of (%) inhibition of SARS-CoV-2 binding to the human host receptor angiotensin converting enzyme-2 after vaccination with BNT162b2 (Pfizer) and ChAdOx1 (AstraZeneca, AZ) vaccines. Rate of change values (RC) express the average change in antibody titers between two time points and were estimated for two consecutive time points: RC M3–M1 and RC M6–M3. Since the percentage inhibition decreases with time, the RC values are negative. However, the absolute numbers are shown in the graph. The error bars correspond to standard deviation, while the asterisk (*) indicates statistically significant differences between the compared groups (*p*-value < 0.05).

In order to further examine how quickly NAbs inhibition decreases after the second vaccination, the term “rate of change” was used. In this study, the units of “time” were considered “days” so that the rate of change reflected the average daily difference in NAbs titers. A visual representation of each RC for the Pfizer and AZ groups is provided in Figure 4.

Because NAbs measurements were taken on three occasions, there are two RC estimates for each vaccine based on the averages of the two time intervals (i.e., M3–M1 and M6–M3). The percentage RC for the M3–M1 interval for Pfizer’s vaccine was 11.0%, while the corresponding value for AZ was 28.4% (significant difference: *p*-value < 0.001). This result suggests that the elimination rate of NAbs is higher for the AZ vaccine than Pfizer’s. Additionally, for the next trimester (M6–M3), the elimination rate for AZ (21.1%) remains higher than for Pfizer (14.6%). The latter was not found to be statistically significant (*p* = 0.803). It is worth noting that the elimination rate for the AZ vaccine decreases over time, while it increases for Pfizer. However, in both cases, the changes were not found to be statistically significant.

The effect of many other factors on NAbs was also examined. The subjects’ age, medical history (i.e., comorbidities), BMI, and medications were examined to determine whether they affected antibody levels on each day or if they could affect the reported reduction after M1. Due to the small age range of the participants in this study, no age-related changes were observed. In addition, none of the other covariates had a statistically significant effect on NAbs levels.

## 4. Discussion

Although real-world observational studies have confirmed the effectiveness of BNT162b2 and AZD1222 vaccines against COVID-19 [22], the durability of protection has been questioned due to the evidence of waning immunity, the emergence of new SARS-CoV-2 variants and the reports of breakthrough infections [12,13,14,15,16,17,20]. Both vaccines induce immunological memory against the S protein of SARS-CoV-2. The spike (S) protein plays a key role in SARS-CoV-2 entry in human cells. The receptor-binding domain (RBD) of the S viral protein binds to the angiotensin-converting enzyme 2 (ACE2) receptors on the surface of human cells and, following the fusion of viral and cellular membranes, SARS-CoV-2 enters human cells [23]. The high affinity of S-RBD to the ACE2 receptor is responsible for the high transmission rate of SARS-CoV-2 and COVID-19 [23]. The manufacture of BNT162b2 is based on a three-lipid nanoparticle (LNP)-formulation encapsulated mRNA platform and induces both high antibody titers and cellular responses against SARS-CoV-2 [23]. It is intramuscularly administered in two doses 3 weeks apart. AZD1222 is based on a chimpanzee adenovirus vector displaying the S protein on its surface and it induces strong humoral responses against SARS-CoV-2 [23]. It is intramuscularly administered at two doses which are 12 weeks apart. For both vaccines, a third booster dose is administered at least 5 months from the second dose, whereas a fourth booster dose may be administered in individuals with comorbidities [19,24,25]. A booster shot has been associated with enhanced and durable antibody responses following vaccination against other viruses, such as the Ebola virus with the ChAd3 adenoviral vector vaccine [26].

In our study, both BNT162b2 and AZD1222 induced strong NAbs responses at 1 month following a two-dose vaccination scheme. Interestingly, NAb activity seemed superior with BNT162b2 compared with AZD1222. A gradual decline in NAbs titers was evident at 3 and 6 months post vaccination with both vaccines. However, the superiority of NAb response with BNT162b2 over AZD1222 remained consistent at all time points examined. Furthermore, the elimination rate of the NAb titer was higher throughout the study period for those vaccinated with AZD1222 compared with BNT162b2. The full-length S protein with the leader sequence of tissue plasminogen activator is presented by the AZD1222 to antigen-presenting cells [27]. The BNT162b2 encodes for the full-length S protein, and it is also stabilized in the prefusion conformation [28]. The differences both in the manufacture process of the two vaccines and in the tertiary structure of the S protein, along with the frequency of the dosing scheme, may be associated with the different pattern of antibody decline over time between AZD1222 and BNT162b2.

Our results are consistent with another recent study including 121 individuals vaccinated with either BNT162b2 (*n* = 36) or AZD1222 (*n* = 85) that assessed the humoral and cellular immunity during a 3-month period following vaccination [29]. The NAbs level was 6-fold higher with BNT162b2 compared with AZD1222 at 2 weeks after the second vaccine shot. Interestingly, the NAbs titers with BNT162b2 were similar to the NAbs titers at 1 month post symptomatic COVID-19 among 18 convalescent individuals. The SARS-CoV-2 S1 protein-specific IgG antibody titers were also higher post vaccination with BNT162b2 compared with AZD1222, but humoral responses declined over time in both cases [29]. Furthermore, individuals vaccinated with BNT162b2 show persistent T-cell responses even at 3 months post vaccination, whereas the T-cell responses were attenuated in those vaccinated with AZD1222 [29]. Differences in the cellular responses between the two vaccine types may be implicated in the distinct kinetic profile of antibody decline over time.

Importantly, the two-dose vaccine effectiveness against the Delta variant of SARS-CoV-2 remains high for both BNT162b2 (94%) and AZD1222 (88%) [16,30]. This is also reflected by the low viral load of breakthrough infections among recently vaccinated individuals [31]. However, the corresponding percentages may fall to 42–57% at 4–6 months following vaccination [14,30,32]. Nevertheless, the protection against severe COVID-19 and associated hospitalization remains substantially high for both BNT162b2 (77–93%) and AZD1222 (70%) [14,30,32]. Overall, data from both randomized control trials and observational studies advocate for a persistent but attenuated level of protection against COVID-19 at 6 months following vaccination with BNT162b2 [15,17,33,34,35,36,37,38,39]. It should be underlined that memory B- and T-cells may persist despite the decrease in anti-spike, anti–receptor binding domain antibodies and NAbs against SARS-CoV-2 [40]. This immune memory may be responsible for the protection against severe COVID-19 upon infection with SARS-CoV-2 [40]. Relevant data on the durability of humoral responses following vaccination with AZD1222 are scarce in the literature.

Booster vaccination with a third dose of BNT162b2 at 5 months after the second vaccine shot restores the robust humoral response and enhances the level of protection against severe COVID-19 and mortality due to COVID-19 compared with those vaccinated with two BNT162b2 doses 5 months before or earlier [41,42,43]. Improved antibody responses and neutralizing activity with a booster COVID-19 vaccine has been also reported in individuals who have previously been vaccinated with two doses of AZD1222 at least 70 days prior [24]. Therefore, booster vaccination may be considered even before the 5-month time interval following the last vaccine shot for those vaccinated with AZD1222, also taking into consideration the results of our study.

A limitation of this study is the limited sample size, which may make it difficult to examine certain pathophysiological conditions, such as autoimmune diseases known to affect the immune response. Among the five currently approved vaccines in Europe (Comirnaty (BioNTech/Pfizer), Nuvaxovid (Novavax), Spikevax (Moderna), Vaxzevria (AstraZeneca) and COVID-19 Vaccine Janssen), we only evaluated Cominarty and Vaxzevria because they are the most widely administered in Greece. Furthermore, due to initial limitations in the age range of vaccinated individuals with AZD1222 according to the availability of data from the initial clinical trials of the vaccine, the age range of this analysis is narrow and possible age-specific variations in NAbs kinetics could not be determined. Therefore, the subgroup analyses performed in this study should be considered exploratory. A further analysis of the new COVID-19 cases and COVID-19-related deaths over time would provide a more complete insight into the duration of vaccine protection.

Regarding the role of gender in the multiple comparisons in the present analysis, it should be noted that men and women in the Pfizer group had an unequal sample size (36.1% vs. 63.9%). However, this imbalance did not affect the analysis as the focus of the comparisons in this study was on the type of vaccine. Therefore, subjects of the same sex were compared with the two vaccines.

## 5. Conclusions

This study provided evidence for a consistent decline in NAbs levels for both BNT162b2 and AZD1222 from one month to six months post vaccination. BNT162b2 results in more robust NAbs responses that decline over time more slowly than AZD1222. It has to be underlined that the medical compatibility of each individual should be taken into consideration, in addition to the effectiveness of each vaccine, in order to choose the vaccine type. However, a booster dose is necessary to achieve an adequate neutralizing response against the Omicron variant [21]. Our results may inform public health policies regarding the timing of the administration of booster COVID-19 vaccine shots, also taking into consideration the emergence and the prevalence of new variants of concern including Omicron.

## Figures and Tables

**Table 1:** Number of individuals who received the BNT162b2 or ChAdOx1 vaccine and their characteristics.

Characteristics	BNT162b2	ChAdOx1
Sample size	83	199
Gender		
Men	30 (36.1%)	56 (47.1%)
Women	53 (63.9%)	63 (52.9%)
Age (median)	61.0	61.5
Body mass index (median)	26.7	26.6
Underweight (*n*, %)	0 (%)	0 (0%)
Normal weight (*n*, %)	28 (33.7%)	37 (31.1%)
Overweight (*n*, %)	40 (48.2%)	45 (37.8%)
Obese (*n*, %)	15 (18.1%)	37 (31.1%)

## Data Availability

The data presented in this study are available on request from the corresponding author.
